# Effectiveness of an ultrasound basic cancer training program through on-site training and virtual case discussions in rural Tanzania: a proof-of-concept study

**DOI:** 10.3332/ecancer.2024.1722

**Published:** 2024-06-28

**Authors:** Johannes Matthias Weimer, Eva Kuhn, Michael Ludwig, Goodluck Lincoln Malle, Godfrid Kapipi, Valentin Sebastian Schäfer, Adnan Sadiq, Oliver Henke

**Affiliations:** 1 Rudolf -Frey Learning Clinic, University Medical Centre of the Johannes Gutenberg-University Mainz, Mainz 55131, Germany; 2 Section Global Health, Institute of Hygiene and Public Health, University Hospital Bonn, Bonn 53127, Germany; 3 Department of Internal Medicine I, Hospital of the German Armed Forces, Berlin 10115, Germany; 4 Marangu Lutheran Hospital, PO Box 107, Marangu, Tanzania; 5 Department of Rheumatology and Clinical Immunology, Clinic of Internal Medicine III, University Hospital Bonn, Bonn, Germany; 6 Kilimanjaro Christian Medical University College, Moshi 2240, Tanzania

**Keywords:** POCUS, online tumorboard, curriculum development, LMIC

## Abstract

**Introduction:**

Cancer rates are rising in low- and middle-income countries. While point-of-care ultrasound is now available globally and could serve to mitigate against this rise, its use in diagnosing cancers is inconsistent in lower-resourced healthcare contexts. This proof-of-concept study investigates the feasibility of an ultrasound training concept in a low-resource setting. It evaluates whether this educational concept led to improved knowledge and application of ultrasound diagnostics, cancer screening and staging and patient care.

**Material and methods:**

The curriculum was developed through expert exchange and is based on the World Health Organisation’s Manual of Diagnostic Ultrasound. It consisted of two didactic components: an on-site training phase across 5 days for a total of 24 hours, and a digital follow-up phase involving the meeting of a bi-weekly tumor board online. The learning objectives of the on-site training were normal imaging and recognition of common pathologies of the abdominal organs, vessels, lymph nodes, female breasts and lungs. The virtual tumour boards met to discuss cases and ultrasound findings, thus aiding continuing professional development after the training sessions had concluded. The face-to-face course component was accompanied by tests given before and after training as well as an evaluation sheet (Likert-scale with 1 = ‘completely/very good’ and 7 ‘not at all/very poor’).

**Results:**

Of 20 participants from a rural hospital in Tanzania, a total of 16 were included in the analysis (clinical officers *n* = 6; medical officers *n* = 10). A significant increase in knowledge (*p* < 0.01) was measured both in the subjective self-assessment and in the theoretical competence tests. In multivariate linear regression, the status ‘medical officers yes’ (*β* = 5.4; *p* = 0.04) had a significant influence on theory test results at T2. During the 24 virtual tumour board meetings, 28 cases were discussed and a continuous improvement in image acquisition quality was observed.

**Conclusion:**

The ultrasound education concept comes with a sustainable increase in clinical competence and improved oncological ultrasound screening locally. There is potential for the transfer of the concept to other locations, which can be explored in the future.

## Introduction

Nations with low human development indices are projected to experience a twofold increase in the incidence of cancers between 2020 and 2040 [1]. Low- and middle-income countries (LMICs) already account for more than 60% of global cancer incidence and 70% of global cancer-related mortality [2], and the risk of dying from cancer is consequently higher in LMICs than in high-income countries (HICs). To address this growing burden in cancer diseases, health systems need to adapt and focus more on cancer and other non-communicable diseases (NCDs) in the future.

However, this transition is currently hindered by LMICs having generally weak healthcare systems with limited expertise in NCDs.

A predominant challenge, especially in Sub-Saharan African countries, is the late-stage presentation of patients [3]. Up to 80% of newly diagnosed cancer patients are identified only in stages III or IV of cancer progression [4, 5]. However, healthcare systems themselves can cause this delay in diagnosing and treating patients: specialised treatment centres are often chronically understaffed and limited in number [5]. In a recent multi-centre study from Tanzania and Uganda on children and adolescents with lymphoma, healthcare systems caused two-thirds of the delays between the observation of first symptoms to the initiation of treatment [6].

Despite its population of approximately 60 million people [7], Tanzania has just three public cancer treatment facilities and two private hospitals [8] to provide oncologic services for the whole country. Tanzanian policy and public health strategy have recognised these deficiencies. The Tanzanian government’s National Cancer Control Strategy 2016–2022 determined ‘early detection’ as crucial in mitigating cancer risks to its population [9]. The National Cancer Treatment Guidelines list ultrasonography as the most important diagnostic tool in achieving this early detection [10]. However, limited oncology training of healthcare professionals as well as delays in diagnostic procedures and referrals to specialised centres impede achieving this aim in Tanzania and beyond [3].

It is not only Tanzania that has recognised the importance of point-of-care ultrasound (POCUS) [11, 12] for screening, staging and follow-up diagnostics across a range of cancers [10, 13–15]. POCUS is now used globally and offers numerous advantages to healthcare systems and the patients they serve. As a non-invasive, portable, highly reproducible and relatively cost-effective method, POCUS surpasses other traditional imagining modalities that pose the risk of ionizing radiation, such as X-ray or computed tomography [12, 16–19]. Nonetheless, elsewhere as in Tanzania, a lack of training is a barrier to the broader use of sonography [20–23]. Moreover, curricula developed in the Global North do not account for the healthcare system and provision challenges in Tanzania and other Sub-Saharan African countries [19, 24–26]. Unfortunately, efforts to build capacity in context-specific training are often left unevaluated, or else the evaluation is not shared publicly [12, 19]. Particularly for the improvement of cancer staging, few POCUS training concepts exist [12, 19, 27].

For this reason, this proof-of-concept study aims to demonstrate the feasibility of incorporating POCUS skills, delivered through a 1-week course and complemented by bi-weekly virtual tumour boards, into a clinical setting in an LMIC. The overarching goal is to expedite the referral time to a specialised centre. The study therefore first describes the development of an adapted ultrasound curriculum for LMIC with a focus on cancer diseases. Secondly, we outline the execution of the course, including pre- and post-tests, and finally, we report ultrasound findings of the virtual case discussions as a proxy for clinical outcomes.

## Methods

### Study design and recruitment of participants

This proof-of-concept study was designed as an observational trial [28]. The study was part of a 2022–2024 hospital partnership project between the University Hospital Bonn, Global Health Section in Germany and Marangu Lutheran Hospital (MLH) in Tanzania. A curriculum was developed through an expert exchange and implemented at MLH. Participants were medical and clinical officers from MLH and Kilimanjaro Christian Medical Centre (KCMC), the university hospital and referral centre respectively in Northern Tanzania.

Written evaluations and assessments of theoretical learning outcomes were conducted at two-time points (T1 and T2) to measure the subjective and objective acquisition of skills and acceptance of the training curriculum [29]. The inclusion criteria of individual medical/clinical officers were the consent to participate in the study and the completion of the evaluations and theoretical learning outcomes. In addition, virtual tumour boards were used to regularly evaluate ultrasound findings at additional time points.

**Figure 1.**
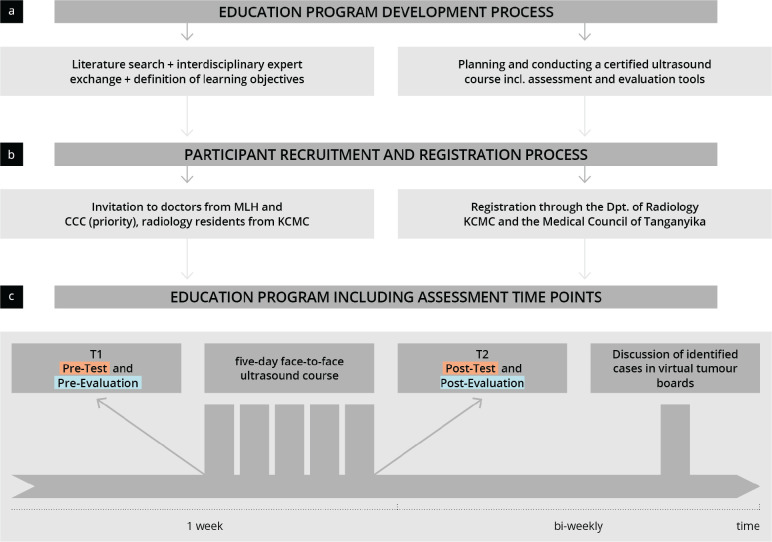
Visualisation of the study procedure. The flow chart shows the education program development process (a), the participant recruitment and registration process (b) and the implementation of the education program (c); MLH = Marangu Lutheran Hospital; KCMC = Kilimanjaro Christian Medical Centre; CCC = Cancer Care Centre at KCMC.

The primary endpoint was defined as the subjective and objective increase in competence as measured using pre- and post-tests. Secondary endpoints refer to the acceptance and satisfaction of the developed training concept as well as the improvement of the documentation of ultrasound findings within the virtual tumour boards. Figure 1 outlines the procedure of the study including the study data collection dates.

### Setting

MLH is a faith-based district hospital in the Kilimanjaro region of Northern Tanzania, serving a population of 300,000 in a broad catchment area. The internal medicine, surgery, obstetrics and paediatrics wards have 80 beds and 80 employees, including four medical officers and five clinical officers, but no specialists. MLH offers both inpatient and outpatient care. In addition to a basic clinical laboratory, a diagnostic X-ray machine is available and one ultrasound machine is operated by a single radiographer. Oncology patients are referred to the Cancer Care Centre of the KCMC.

### Development and implementation of the course curriculum

The course curriculum was designed through a process of close collaboration between Tanzanian and German medical doctors with expertise in sonography, radiology and oncology. Supplement 1 shows the schedule and the learning content of the ultrasound course. Both were based on the World Health Organisation Ultrasound Manual [30].

Specialists communicated via e-mail and online calls to jointly define the curriculum and a course schedule that balanced theory and practical training. The final curriculum was certified by the European Federation of Societies for Ultrasound in Medicine and Biology, and the course was also accredited by the Medical Council of Tanganyika (the board of physicians for Tanzania´s mainland), which grants continuous professional development credits for attendance.

Two middle-class ultrasound devices (ACUSON NX3^©^ SIEMENS Healthineers, Germany) and two pocket ultrasound devices (LUMIFY^©^ PHILIPS, Netherlands) were used for practical training. This training took place in small groups with a maximum of six participants per tutor.

The course was led by two German ultrasound specialists certified by the German Society for Ultrasound in Medicine (DEGUM), along with a radiology specialist from Tanzania.

Apart from the medical/clinical officers from MLH, the course welcomed participation from medical/clinical officers at KCMC to foster a broader impact and promote future collaboration, including network building and other mutual learning experiences.

### Evaluation of the ultrasound training and course curriculum

Participants completed evaluations and written tests (Supplement 2) both before and after the course (T1 and T2, respectively). The pre-course evaluation questionnaire, the pre- and post-tests, and the examined teaching content were developed through a combination of ultrasound and educationalist expert consensus and the recommendations of professional associations.

The learning objectives of the course served as the basis for the questions in the pre- and post-quizzes. The questions used in the test related to ‘ultrasound basics’ (anatomical directions, artefacts, image orientation, technical principles and transducers) and ‘standard views’ (vessels, pancreas, liver, kidney and pelvic organs) [31]. Answers were required to be given as short free texts rather than more typical multiple-choice questions [32]. In total, 33 points could be achieved (12 for ‘basics’ and 21 for ‘standard views’). Some of the test questions (‘standard views’) were adapted from other DEGUM-certified courses and based on DEGUM recommendations [31].

The pre-course evaluation questionnaire asked for demographic information about the participants, previous experience, familiarity with ultrasound equipment, familiarity with self-learning methods and pre-course skills, using a Likert scale (with 1 = ‘completely/very good’ and 7 ‘not at all/very poor’). The post-course questionnaire included an evaluation of the course by the participants.

### Virtual case discussions in the virtual tumour boards

After the face-to-face course, participants received regular fortnightly virtual case discussions via Zoom^®^. In these sessions of approximately 60 minutes each, one to two cases were presented and discussed. A case presentation consisted of a summary of the patient’s medical history and clinical presentation, followed by a presentation of digital ultrasound images and video loops. The case presentation, conducted by a clinical or medical officer from MLH, was followed by a review of the ultrasound findings and a conclusion about the likely clinical diagnoses and further patient management. The health insurance status and financial capabilities of the patients were considered in decision-making to provide the patient with the most resource-efficient and effective therapy and to avoid financial catastrophic losses. All data, including ultrasound images and videos, were stored in the online platform SATMED^®^ to fulfil European regulations of medical data handling. Patient identifiers were not captured and only the MHL team could assign the data to the respective patient. Besides the case discussions themselves, these sessions implemented a positive feedback loop for the teaching of image settings and documentation.

### Statistical analysis

After analysis of the pre- and post-tests, data was entered into an MS Excel database before being transferred to RStudio (RStudio Team. RStudio: Integrated Development for R. 2020.) and R 4.0.3 (R Foundation for Statistical Computing. A Language and Environment for Statistical Computing, R. 2020). Then, descriptive, explorative and inferential statistical analyses were carried out. The metric scales were tested for normal distribution using the Shapiro-Wilk test. For the scales that were not normally distributed, the non-parametric Wilcoxon-Mann-Whitney test was applied instead of the parametric *t*-test or Welch Two Sample *T*-test. In addition, a multivariate linear regression model was produced to compare the influence of individual factors. *p*-values less than 0.05 were considered statistically significant.

## Results

### Study population

Of 20 participants, the fully completed pre- and post-surveys of 16 were included in the analysis. Table 1 and Supplement 3 give an overview of the participants and their previous ultrasound experience. More than 62.5% (*n* = 10) of the participants were medical officers and 37.5% (*n* = 6) were clinical officers. Overall, 50% of the participants stated that they had already performed more than ten independent ultrasound examinations and that they mainly (56.3%) learned independently using ‘online videos’. The majority (81.3%) of the participants have access to a sonography device in the hospital.

### Subjective results: self-assessment and course evaluation

**Figure 2.**
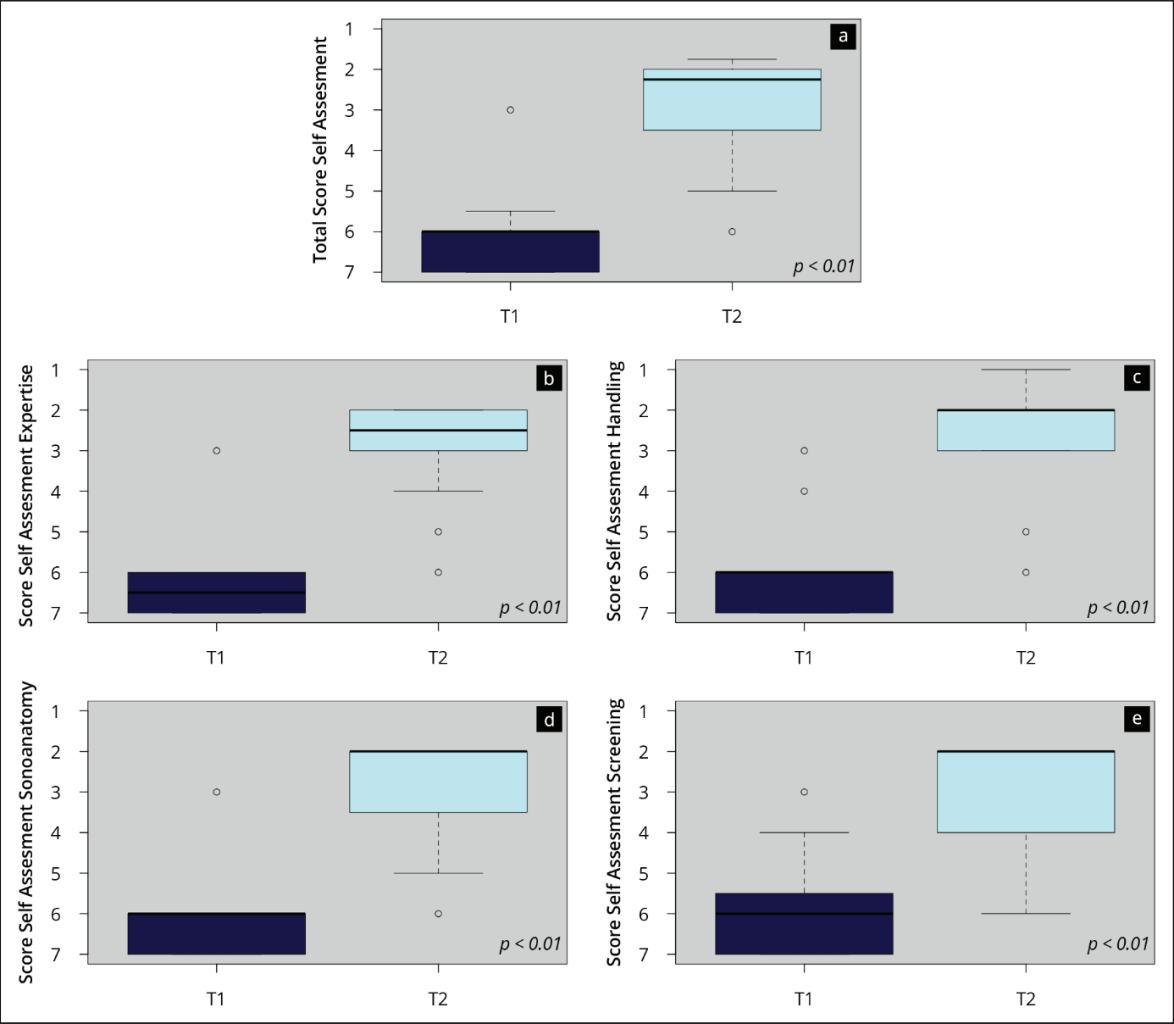
Results of the competence self-assessment. The boxplots visualise the total score (a) and the scores of the subcategories of the self-assessment (b–e) at the time points T1 (beginning) and T2 (end of the onsite course).

Figure 2 and Supplement 4 show the results of the subjective competence assessment before (T1) and after (T2) the ultrasound course as well as the results of the course evaluation. After completing the course, the participants indicated a significant (*p* < 0.01) increase in skill level in both the total self-assessment score (T1: 6.1 ± 1.0 versus T2 2.8 ± 1.2) and scores of all sub-areas (‘sonoanatomy’, ‘screening’, ‘transducer handling’ and ‘ultrasound expertise’). Transducer handling especially was evaluated in higher scale ranges. Also, the ‘instructors’ (1.1 ± 0.3), ‘teaching material’ (1.3 ± 0.5) and ‘course’ (1.25 ± 0.5) were rated by the participants in very positive scale ranges (1–1.5).

### Objective results- theory assessment

**Figure 3.**
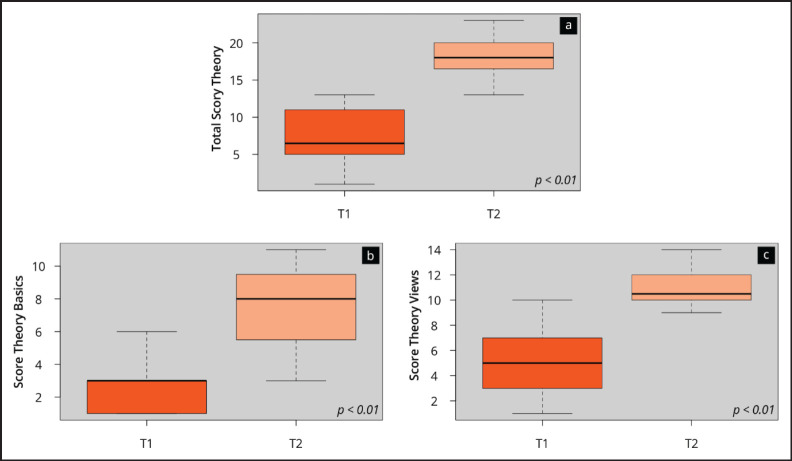
Results of the objective competence assessment. The boxplots visualise the total score (a) and the scores of the subcategories of the theory test assessment (b–e) at the time points T1 (beginning) and T2 (end of the onsite course).

Figure 3 visualises the results of the theory test at the beginning (T1) and at the end (T2) of the ultrasound course. It shows a significant (*p* < 0.01) increase in ultrasound competence on the objective theory test (T1: 7.5 ± 3.7 versus T2 18.4 ± 3.0). The improvement was seen in all subcategories of the assessment.

### Influencing factors and correlations

The possible influence of prerequisites, including professional status and ultrasound experience, on the subjective (self-rating) and/or objective (theory test) ultrasound competence assessment and the measured competence improvements, were also analysed separately and are shown in Supplement 5. Significant differences were only found in a subgroup analysis of the theoretical test results for professional status, showing that the theoretical improvement of competence after the course was significantly higher for the medical officers compared to the clinical officers (*p* < 0.01). In the multivariate linear regression analysis of the results of the theory tests at T1 and T2, ‘previous ultrasound experience/course yes’, ‘status medical officer yes’, ‘sonography workplace yes’ and ‘sex’ were defined as possible influencing factors. Again, the ‘medical officer yes’ factor (*β* = 5.4; *p* = 0.04) had a significant influence on the theory test results in T2.

### Virtual case discussion

**Figure 4.**
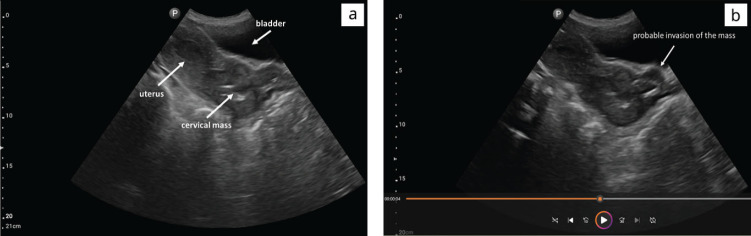
B-Modus sagittal plane of a female pelvis. (a) Ultrasound images show the cervical mass with probable extension beyond cervical margins (b).

Following the practical course, 24 virtual tumour boards have been conducted to date, in which 28 cases were discussed. The feedback loop on image settings and documentation has led to continuous improvement of picture quality (indentation depth, gain and frequency) and clip recording. Figure 4 and Supplement 6 display one of the cases discussed during a tumour board.

## Discussion

This study aims to detect cancer at an early stage and, at least, earlier than currently. For this, clinical and medical officers in Tanzania have been empowered to use POCUS diagnostics and participate in fortnightly online case discussions.

The results of this pilot show that the participants of a setting-adapted ultrasound course achieved a higher level of competency in ultrasound diagnostics by the end of the course. In addition, the training concept was received exceptionally positively.

Furthermore, there has been a marked improvement in ultrasound image settings and documentation over time, as evidenced by the online case discussions. Eventually, some patients’ cancers were identified at likely early(ier) stages, and thus the possibility of curative treatments was preserved.

### Developing adapted ultrasound curricula for and with healthcare providers in LMIC

Currently, a major barrier to the use of ultrasound in the clinical practices of developing countries is the lack of training in general and the absence of specific oncology curricula for primary care [20, 21, 23, 26]. Therefore, ultrasound training concepts based on local patterns of disease are imperative [19, 33]. This adapted ultrasound course, developed for and with healthcare providers in Tanzania, was positively evaluated by all participants. The overall improvement of the participants’ competencies is in line with the findings of similar studies [25, 27, 34]. The inclusion of clinical officers into the course comes with two potential long-term benefits: in contrast to medical officers, clinical officers usually spend their entire professional career in the same hospital. This reduces the loss of knowledge in rural areas due to staff movements and increases the likelihood that knowledge is passed on amongst hospital staff. However, knowledge transfer between staff cannot be taken for granted and must be advocated for in Sub-Saharan African medical settings [35, 36]. Strategies must be developed for a so-called ‘train-the-trainer’ model or other approaches. Still, the inclusion of clinical officers into the course concept contributes to the sustainability of knowledge transfer and can mitigate against skills shortages by introducing POCUS for identifying cancer at likely early(ier) stages in non-specialised, district-level hospitals [33].

Medical officers had a greater gain in competency than clinical officers, which is consistent with results from comparative studies [25, 33, 34]. This effect may be explained by potential language barriers, as the course was given in English, or by general differences in pre-training and levels of previous education. If future training similarly maintains the focus on clinical officers, training sessions should account for this finding by giving translations into local languages. In addition, ultrasound training should be considered for inclusion in the clinical officers’ curriculum [37].

With its focus on the staff of non-specialised district-level hospitals and early(ier) detection of cancer diseases, this training concept can be distinguished from other training programs that mainly focus on the use of POCUS in emergencies in cardiology, gynaecology and obstetrics [12, 25, 34, 38–41]. Moreover, and in contrast to other courses, the concept is adapted to the local patterns of cancer diseases in Tanzania and remains ongoing through online meetings. The combination of online case discussions addresses the lack of specialist knowledge in this clinical context and delivers continuous medical education. Such support ensures a project’s sustainability, maintains the trainees’ motivation and promotes high levels of capacity-building.

### Virtual case discussions and decision-making

The introduction of virtual case discussions has significantly changed medical decision-making, particularly in resource-limited environments [42, 43]. In our ultrasound training program, virtual tumour boards for case discussion have offered several pragmatic benefits as well as the overcoming of geographical constraints [44].

One primary benefit of virtual discussions is the ability to access global expertise, which can mitigate local absences [42, 43]. Online connectivity facilitates the easy sharing of knowledge and insights among clinicians, thereby enabling prompt and well-informed decisions for patient care [45, 46] and eliminating patient travel for specialised consultations [47].

Our study's outcomes further underscore the usefulness of pairing ultrasound training with virtual case discussions. By integrating remote case presentations with video loops from ultrasound examinations, we were able to identify early-stage cancer cases, eventually resulting in improved survival prospects. This pivotal finding emphasizes the potential of this approach in promoting the early detection of cancers and subsequent patient management. However, as Kabukye *et al* [45] displayed in their scoping review, the integration of virtual tumour boards (and other telemedicine technologies) in Africa into the clinical routine is still facing resistance.

Additionally, our employment of online platforms like Zoom has demonstrated satisfactory video quality, enabling detailed discussions and collaborative analysis of ultrasound findings.

Our research is valuable in demonstrating a unique cross-border collaboration between an HICs university hospital and a low-level hospital in a low-income national setting. This partnership highlights the possibility of using telemedicine to bridge gaps in healthcare provision, offering providers in resource-limited environments access to specialised knowledge and training.

In conclusion, the integration of virtual case discussions into our ultrasound training program demonstrates how cancer-patient care within resource-limited contexts might be improved. Our implementation of context-specific training coupled with digital expertise shows the tangible benefits of cross-border collaboration in promoting early cancer diagnoses and optimal patient management. As technology advances, this approach holds the potential for wider transformative applications in global healthcare delivery.

## Limitations

Our study has some limitations. First, the number of participants was relatively low for statistical analysis. However, as this study is a proof of concept, we believe that applying statistical methods to the sample was appropriate. Second, there was no control group and therefore randomisation was not possible. Third, although validated ultrasound sections were tested in the theoretical test, the test was not fully validated and no practical tests were administered. For the practical implementation, it should be noted that the main aim of this publication is a proof of concept for a combination of POCUS training and regular virtual case discussions. There are initial indications that this can lead to detecting potential early(ier) stages of cancer. Nonetheless, this does not replace the proper staging of cancer at a specialised facility. The focus is exclusively on timely referral to a tertiary centre. Regardless of the primary centre's findings, the specialist centre would then carry out further staging diagnoses, such as computed tomography scans. In light of potential harm to the patient and their health in case of replacement of staging, the limitations of applying basic POCUS on the primary healthcare level are frequently addressed in the virtual case discussions and cannot be pointed out enough.

## Future perspectives

Besides these limitations, the concept has proven to be feasible and straightforward to implement in daily hospital routines.

As the programme progressed, an on-site follow-up, designed as a refresher course, was held 1 year after the basic course. This second course is not the subject of this proof of concept. In addition, exchange programmes with shadowing took place to improve the ultrasound skills of the participants and the local structure of conducting ultrasound investigations.

Since the participants were familiar with the use and handling of digital teaching media, the training concept could be expanded in the future to include a digital preparation phase as part of a blended learning approach. Such an approach could result in further increases in competence or align attainment levels for preliminary training; equally, a digital tool might also save costs [48–50]. In the future, such training concepts should also be accredited by local professional societies [51].

The practical training within the concept deliberately took place not only on mid-range devices but also on so-called handheld ultrasound devices. As a result of further technical development, these devices also offer the possibility of generating adequate recordings and are good alternatives to conventional devices, not least because they are inexpensive to purchase [14, 52–55]. These devices could be used more widely for screening in the future.

As the training concept develops, the online case discussions should become the responsibility of the respective regional referral hospital to ensure implementation into the Tanzanian healthcare structure.

For the training to be delivered in the future by local experts using a 'train the trainer' approach, a basis for 'trainer certification' would need to be established, which would require further training (including improvement of didactic skills). Standardised assessment methods could be used in the future for a more objective assessment of the images shown in online case discussions [56].

## Conclusion

Our work intends to serve as an example of implementation research in the field of global oncology. This study demonstrates the concrete advantages of international collaboration in the development and implementation of an ultrasound training concept for early cancer detection. A unique feature of our research is the cross-border collaboration between a university hospital in an HIC and a primary care hospital in an LMIC. The combination of traditional on-site training with virtual support represents an innovative approach to implementing educational approaches in a non-specialised district hospital setting. This study highlights the importance of using telemedicine and digital education to bridge gaps in care and provide healthcare providers in resource-poor settings access to specialist knowledge.

Adapted, short-term training in POCUS for LMIC is possible and leads to healthcare personnel being empowered in specialised medical fields. This may contribute to mitigate the shortage of specialists and reduce the workload for referral hospitals. Regular online case discussions with specialists ensure quality of care. Finally, decisions on the referral of cancer patients in early stages can be made promptly, thus contributing to better treatment outcomes.

A transfer of the training concept to other non-specialised district hospitals through a hub-and-spoke model is plausible and should be targeted in the future to enable ultrasound diagnoses more comprehensively.

## Conflicts of interest

The authors declare no conflicts of interest.

## Funding

This programme is part of the GIZ (Gesellschaft für Internationale Zusammenarbeit) & EKFS (Else Kröner-Fresenius Stiftung) funded ‘Hospital Partnership Programme’.

## Consent for publication

Informed consent for publication of identifying information/images was obtained from all subjects and/or their legal guardians.

## Author contributions

Conceptualisation: JMW, EK, ML, AS and OH; methodology and software: JMW, ML and OH; validation: JMW, EK, ML, GLM, GK, AS and OH; formal analysis: JMW; investigation: JMW, EK, ML, OH; resources: EK, ML, AS and OH; data curation: JMW, ML, GLM, GK, AS and OH; writing – original draft preparation: JMW, EK and OH; writing – review and editing: JMW, EK, ML, GLM, GK, VS, AS and OH; visualisation: JMW; supervision: JMW, EK and OH; project administration: JMW, EK, AS and OH; funding acquisition: EK and OH. All authors have read and agreed to the published version of the manuscript.

## Ethics approval and consent to participate

Given the nature of the evaluation and in accordance with applicable law the approval for the study was waived by the ethics committee of University Hospital Bonn, Germany. All procedures performed in studies involving human participants were in accordance with the ethical standards of the institutional and national research committee and with the 1964 Helsinki declaration and its later amendments or comparable ethical standards. Informed written consent was obtained from all the participants.

## Availability of data and materials

Data cannot be shared publicly because of institutional and national data policy restrictions imposed by the Ethics committee since the data contain potentially identifying study participants’ information. Data are available upon request from the Johannes Gutenberg University Mainz Medical Center (contact via weimer@uni-mainz.de) for researchers who meet the criteria for access to confidential data (please provide the manuscript title with your enquiry).

## Author information

JMW is resident and coordinator of the student ultrasound education programme of the University Medical Center Mainz. He has more than 10 years of experience in the didactic field and has also worked on the topic of ultrasound training methods in his doctoral thesis. He is a member of the DEGUM and GMA.

OH is a medical oncologist and haematologist and associate professor at the University of Bonn, where he leads the working group on ‘Global Oncology’. OH is the project lead of the hospital partnership programme between Bonn and Marangu.

GLM is a medical doctor and in charge of the MLH and cooperation partner of the hospital partnership programme.

## Figures and Tables

**Table 1.** Baseline characteristics of the participants.

Item	Result
Age (Mean ± SD)	36.1 ± 8.1
Sex Female (*n*, (%)) Male (*n*, (%))	5 (31.3) 11 (68.7)
Position Clinical officer (*n*, (%)) Medical officer (*n*, (%))	6 (37.5) 10 (62.5)
Sonography device at the workplace Yes (*n*, (%)) No (*n*, (%))	13 (81.3) 3 (18.7)
Ultrasound course Yes (*n*, (%)) No (*n*, [%]) Ultrasound experience (> 10 examinations**)** Yes (*n*, (%)) No (*n*, (%))	12 (75.0) 4 (25.0) 8 (50.0) 8 (50.0)
Tools for self-study of sonography Yes (*n*, (%)) No (*n*, (%)) Books Yes (*n*, (%)) No (*n*, (%)) E-learning Yes (*n*, (%)) No (*n*, (%)) Online videos Yes (*n*, (%)) No (*n*, (%))	11 (68.7) 5 (31.3) 5 (31.3) 11 (68.7) 1 (6.3) 15( 93.7) 9 (56.3) 7 (43.7)

**Figure:**
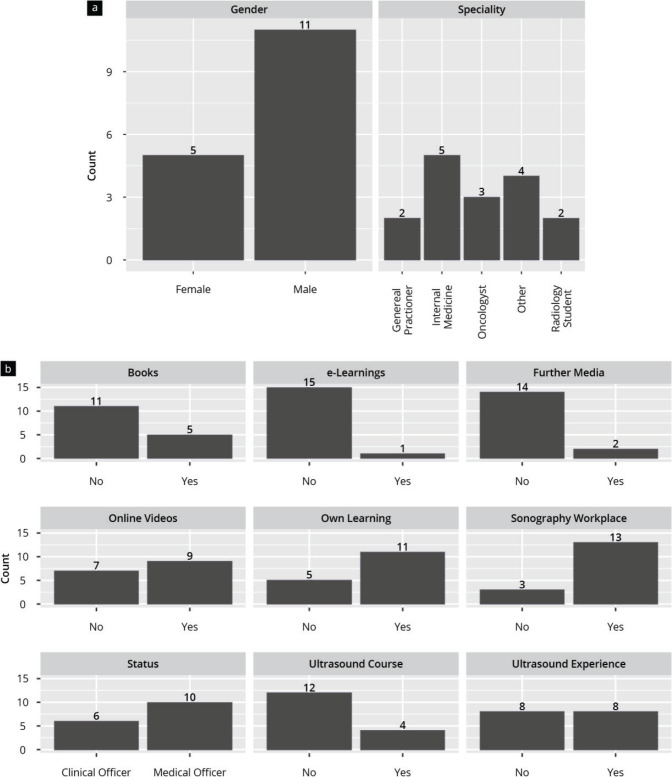
Baseline characteristics of the participants. The histograms show the frequencies (n) of the items from T1.

**Figure:**
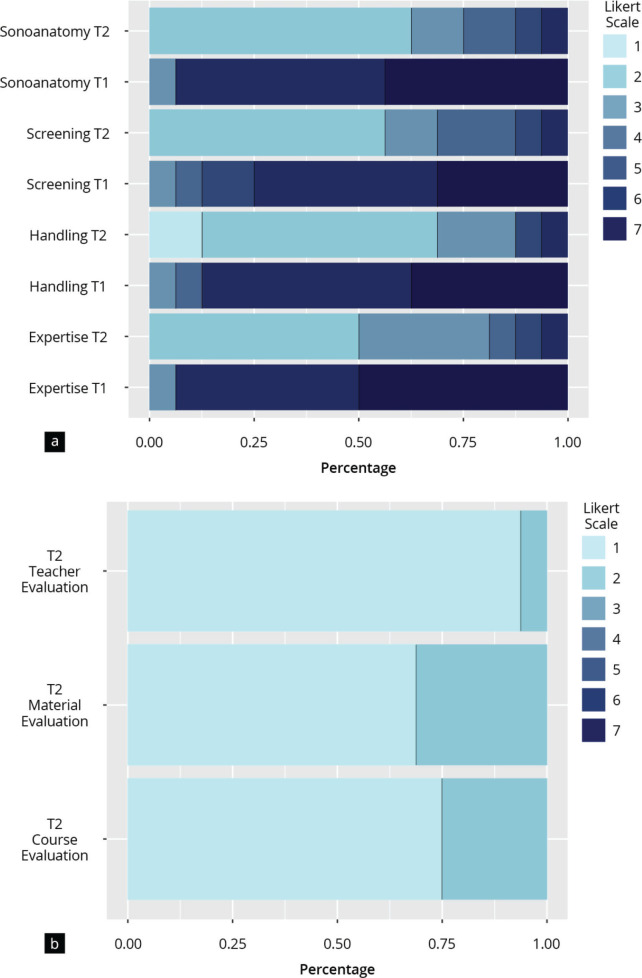
Evaluation results of the competence assessment, the course instructor's performance, the teaching materials, and the course concept both pre-course (T1) and post-course (T2). The graph shows a 7-point Likert scale (1 = “completely”/“very good” and 7 = “not at all”/“very poor”) according to frequencies (percentages).

**Figure:**
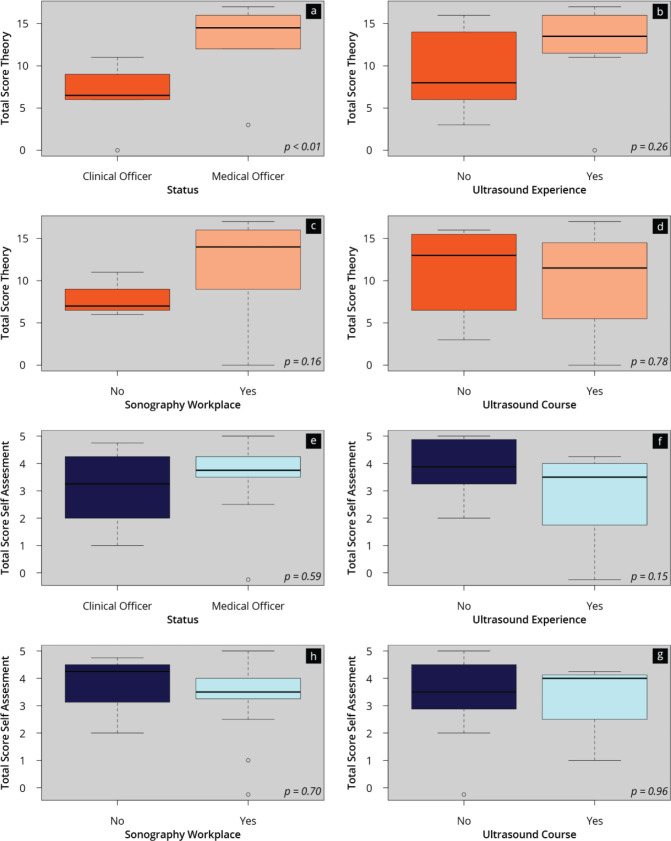
Presentation of possible factors influencing objective (a–d) and subjective (e–g) theoretical competence improvement

## Supplement 1.

**Table:** Schedule + learning content of the ultrasound course

Day	Time	Content
1	09:00 -10:00	Welcome; introduction; and entrance test
1	10:00-10:40	The physical basics of medical ultrasound; sectional planes and documentation
1	10:45 – 12:00	Practical exercises: getting to know the ultrasound systems; buttons/picture acquisition
1	12-00-13:00	Lunch
1	13:00-14:00	Liver anatomy and sectional planes; liver steatosis, hepatitis and cirrhosis; liver portal vein thrombosis
1	14:00 – 16:00	Practical exercises: the liver (orientation, lobes/vessels, sectional planes)
2	09:00-10:00	Liver benign focal lesions; malign focal lesions; quiz
2	10:00 – 12:00	Practical exercise: focal lesions of the liver
2	12-00-13:00	Lunch
2	13:00-14:00	The gallbladder and biliary ducts; the pancreas; free fluid in the pleura and peritoneum
2	14:00 – 16:00	Practical exercises: the gallbladder, bile ducts, pancreas, and pleura
3	09:00-10:00	The spleen; the kidneys; the urinary tract; the retroperitoneum and the vessels
3	10:00 – 12:00	Practical exercises: the spleen, kidney, retroperitoneum and vessels
3	12-00-13:00	Lunch
3	13:00 -14:00	Lymph nodes: basic anatomy; infectious diseases; malignant diseases
3	14:00 – 16:00	Practical exercise: lymph nodes
4	09:00-10:00	Breast cancer and lymph nodes; the prostate gland
4	10:00 – 12:00	Practical exercises: breast cancer, lymph nodes, the prostate
4	12-00-13:00	Lunch
4	13:00 – 13:30	Ultrasound-guided punctions
4	13:30 – 15:00	Practical exercise: ultrasound-guided punctions
4	15:00-16:00	Final test; certificates; farewell
5	09:00 – 12:00	Facultative practical exercise

## Supplement 2: Universitats Kinikum Bonn Evaluation and Quiz

**Questionnaire Precourse**

**A) Personal Data**

1. Please enter your code: __________

2. Age: __________ years old

3. Please indicate your gender: □ m □ f

4. Please indicate your current status:

□ medical officer □ clinical officer/assistant medical officer

5. Please indicate your current specialty, if any:

____________________

**B) Previous experiences and ultrasound equipment**

1. Have your ever preformed an ultrasound examination yourself?

□ yes □ no

If yes, how many times?: __________ times

2. Do you currently have the possibility to use a sonography device at your workplace?

□ yes □ no

3. Have you ever attended a course on ultrasound?

4.

□ yes □ no

If so, what was the time frame of that specific course?

____________________ hours

5. Have you tried to learn sonography on your own so far?

□ yes □ no

If so, which media did you use? (Multiple selection is possible)

□ books

□ online Videos (e. g. YouTube)

□ E-Learnings

□ further media:__________

6. Pre-course skills:

**Table:**

	I have the necessary knowledge and skills related to	Completely				Not at all		
5.1	expertise	□	□	□	□	□	□	□
5.2	Device operation + transducer handling	□	□	□	□	□	□	□
5.5	sono-anatomy	□	□	□	□	□	□	□
5.7	the organ assessment and screening	□	□	□	□	□	□	□

By participating in this course, I hope to:

＿＿＿

**BASICS**

1. Which artefacts are marked by the arrows?

2. Label the image with the correct anatomical directions **(cranial, caudal, ventral, dorsal)** What is the section? Pay attention to the attached transducer image.

3. Please complete the following sentence: The higher the frequency, the __________ the depth of penetration.

4. Which transducers are shown in the following?:

**STANDARD Views**

Which structures are marked in the picture? Describe as precisely as possible!

**Postcourse Questionnaire**

1. Skills after course participation:

**Table:**

	I have the necessray knowledge and skills related to	Completely				Not at all		
5.1	expertise	□	□	□	□	□	□	□
5.2	Device operation + transducer handling	□	□	□	□	□	□	□
5.5	sono-anatomy	□	□	□	□	□	□	□
5.7	the organ assessment and screening	□	□	□	□	□	□	□

By participating in this course, I hope to:

＿＿＿

To what extent do they evaluate the course in terms of:

- Organization+ concept of the face-to-face event

- Achievement of the learning objectives

- Time approach + relationship between theory and practice

How satisfied with the teaching materials are you regarding:

- PowerPoint presentations+ ultrasound equipment

- Pathology presentations+ case studies

- Learning posters

How satisfied were you with the team of trainers in terms of practical and didactic skills?

Individual feedback

＿＿＿

**BASICS**

1. Which artefacts are marked by the arrows?

2. Label the image with the correct anatomical directions **(cranial, caudal, right side of patient, left side of patient)** What is the section? Pay attention to the attached transducer image.

1. Please complete the following sentence: The higher the frequency, the ___________ the depth of penetration.

3. Which **transducers** are shown in the following?:

**STANDARD Views**

Which structures are marked in the picture? Describe as precisely as possible!

## Supplement 6.

**Table:** Summary of a virtual case discussion.

**Patient characteristics**	Female, 62 years of age, multipara in menopause, peasant, cash patient (no health insurance)
Complaints	Prolonged vaginal discharge with a foul smell for 1 year. The discharge was whitish yellow in colour with a pus-like appearance and an initially small, but afterwards minimal amount of blood. Three days ago, she started experiencing lower abdominal pain radiating to her lower back, with sensations of a weight pulling from her vaginal canal to the outside of her body. She also had general bodily weakness, headache and dizziness. No fever, no constipation, no loss of weight, no blood in stool, no cough, no chest pain, no difficulties in breathing, no nausea/vomiting.
Medical history	HIV positive, on regular antiretroviral therapy for more than 10 years, no family history of cancer
Clinical examination	Afebrile, conscious, weak, adult female, pale, no oedema, no peripheral palpable lymph nodes. Vital signs BP 128/84 mmHg, pulse rate 77 bpm, temperature 36°C, SpO_2_ 97% at room air. Per abdomen Not distended, moving with respiration, no palpable mass, moderate suprapubic tenderness and normal bowel sounds. Speculum examination Cervical lesion, with a foul smell and bleeding on contact, occupying the whole cervix.
Laboratory investigations	Full blood picture: WBC 12.13/nL, Hb 7.3 g/dL, MCV- 76.9 fL.
Ultrasound findings (Figure 4a and b)	A cervical mass (less than 4 cm) with irregular margins, mixed hypo- and hyperechoic. The mass seems not to invade the surrounding structures, at one location tumour with possible invasion beyond cervix. Liver and kidneys unremarkable. No free fluid in the abdomen. No enlarged lymph nodes in the pelvis or paraaortal.
Recommendation after virtual case discussion	After reviewing the US loops and photos, the likely diagnosis is cervical cancer. Stage is likely localised, either stage I or stage II, though in one US sagittal view to bladder and uterus, an infiltration of the adjunct structures cannot be excluded. Kidneys without hydronephrosis. Refer to KCMC for biopsy. In this early stage, TAH plus pelvic and paraaortic lymphadenectomy is an option, or else referral to ORCI for radiotherapy. In either case, the patient needs to be informed to present promptly to KCMC to avoid delay in diagnosis and treatment.

KCMC = Kilimanjaro Christian Medical Centre, ORCI = Ocean Road Cancer Institute, TAH = total abdominal hysterectomy, US = ultrasound, WBC = white blood count, BP = blood pressure
